# HIV-1 Sub-Subtype A6: Settings for Normalised Identification and Molecular Epidemiology in the Southern Federal District, Russia

**DOI:** 10.3390/v12040475

**Published:** 2020-04-22

**Authors:** Madita Schlösser, Vladimir V. Kartashev, Visa H. Mikkola, Andrey Shemshura, Sergey Saukhat, Dmitriy Kolpakov, Alexandr Suladze, Tatiana Tverdokhlebova, Katharina Hutt, Eva Heger, Elena Knops, Michael Böhm, Veronica Di Cristanziano, Rolf Kaiser, Anders Sönnerborg, Maurizio Zazzi, Marina Bobkova, Saleta Sierra

**Affiliations:** 1 Institute of Virology, Faculty of Medicine and University Hospital of Cologne, University of Cologne, 50935 Cologne, Germany; madita.schloesser@gmx.de (M.S.); visa.h.mikkola@gmail.com (V.H.M.); katharina.hutt@gmail.com (K.H.); eva.heger@uk-koeln.de (E.H.); elena.knops@uk-koeln.de (E.K.); michael.boehm@uk-koeln.de (M.B.); veronica.di-cristanziano@uk-koeln.de (V.D.C.); rolf.kaiser@uk-koeln.de (R.K.); 2 Russian Southern Federal Center for HIV Control, 344000 Rostov-na-Donu, Russia; vkrt@yandex.ru (V.V.K.); dimakolpakov@mail.ru (D.K.); sualrostov@mail.ru (A.S.); rostovniimp@mail.ru (T.T.); 3 Department of Infectious Diseases, Rostov State Medical University, 344022 Rostov-na-Donu, Russia; sauhat@yandex.ru; 4 Martsinovsky Institute of Medical Parasitology, Tropical and Vector Borne Diseases, Sechenov First Moscow State Medical University, 119435 Moscow, Russia; 5 Clinical Center of HIV/AIDS of the Ministry of Health of Krasnodar Region, 350015 Krasnodar, Russia; shemsh@mail.ru; 6 Division of Clinical Microbiology, Department of Laboratory Medicine, Karolinska Institutet, 17177 Stockholm, Sweden; Anders.Sonnerborg@ki.se; 7 Department of Medical Biotechnology, University of Siena, 53100 Siena, Italy; maurizio.zazzi@unisi.it; 8 Department of General Virology, Gamaleya Research Center of Epidemiology and Microbiology, 123098 Moscow, Russia; mrbobkova@mail.ru

**Keywords:** HIV, drug resistance, subtyping, epidemiology, A6, IDU-A, A-FSU, Russia

## Abstract

Russia has one of the largest and fastest growing HIV epidemics. However, epidemiological data are scarce. Sub-subtype A6 is most prevalent in Russia but its identification is challenging. We analysed protease/reverse transcriptase-, integrase-sequences, and epidemiological data from 303 patients to develop a methodology for the systematisation of A6 identification and to describe the HIV epidemiology in the Russian Southern Federal District. Drug consumption (32.0%) and heterosexual contact (27.1%) were the major reported transmission risks. This study successfully established the settings for systematic identification of A6 samples. Low frequency of subtype B (3.3%) and large prevalence of sub-subtype A6 (69.6%) and subtype G (23.4%) were detected. Transmitted PI- (8.8%) and NRTI-resistance (6.4%) were detected in therapy-naive patients. In therapy-experienced patients, 17.3% of the isolates showed resistance to PIs, 50.0% to NRTI, 39.2% to NNRTIs, and 9.5% to INSTIs. Multiresistance was identified in 52 isolates, 40 corresponding to two-class resistance and seven to three-class resistance. Two resistance-associated-mutations significantly associated to sub-subtype A6 samples: A62V_RT_ and G190S_RT_. This study establishes the conditions for a systematic annotation of sub-subtype A6 to normalise epidemiological studies. Accurate knowledge on South Russian epidemiology will allow for the development of efficient regional frameworks for HIV-1 infection management.

## 1. Introduction

Considerable progress in the fight against HIV/AIDS has already been made, with rates of new infections declining in almost all world regions. However, Eastern Europe and Central Asia showed an alarming increase in annual infection rates of 29% between 2010–2018 [1]. Russia, with over 1.16 million estimated infections by mid-2017 [2,3], is also one of the countries accounting for 89% of all new HIV infections worldwide. However accurate, global Russian data are scarce. Significantly, even the basic incidence and prevalence estimates generated by the Joint United Nations Programme on HIV/AIDS (UNAIDS) cannot be released for Russia because of political sensitivities. Existing knowledge is based only on a limited number of publications, mostly restricted to Moscow and St. Petersburg areas [4], and on reports from several Russian administrative agencies, though the latter are only in the Russian language. Launched in 2014, the UNAIDS Fast-Track strategy outlined plans to step up the HIV response in critical world regions and end AIDS as a public health threat by 2030 (https://www.unaids.org/en/resources/documents/2014/JC2686_WAD2014report). The first step for such an approach must be the identification of the human target population(s), which can differ from region to region. This information, together with the molecular characterisation of the circulating viral strains, will allow for the development of an efficient and specific regional frameworks for HIV-1 prevention, treatment, and monitoring.

The current Russian HIV scenario has been shaped by a massive epidemic in Ukraine and Southern Russia in the mid-1990s, which spread a new sub-subtype: the “Russian A1”, also referred to as “IDU-A”, or “A-FSU” [3,5,6,7,8,9,10]. Phylogenetics has shown that the “Russian A1” is different from the African A1, leading to a classification as the sub-subtype A6 within the subtype A [11]. However, detection of A6 is challenging as there is no reference sequence yet, which in turn impedes the systematic identification by any of the HIV subtyping tools currently used in routine diagnostics. Published epidemiological studies reporting A6 samples have used different genomic regions as well as different sequences as a reference [7,8,12,13,14,15], leading to unsystematic annotation in GenBank and Los Alamos databases. Therefore, the A6 prevalence worldwide is largely unexplored.

A unique feature further differentiates the HIV-1 epidemiology in the Southern Federal District from the rest of the country: the high prevalence of subtype G isolates [3,15]. This can be retraced to a clonal infection episode of more than 250 children after exposure to non-sterile needles during the years 1988 and 1989. The index case was a child admitted to Elista hospital, vertically infected by his mother who, in turn, got infected from her husband after military services in Central Africa [3,16,17,18,19].

The aim of this study was to provide a much needed setting for systematic A6 identification as well as comprehensive data on the HIV epidemiology in the Russian Southern Federal District.

## 2. Patients and Methods

### Study Design and Participants

Blood samples and clinical data from all patients attending the Russian Southern Federal Centre for HIV Control (Rostov-na-Donu), Rostov State Medical University Hospital and the Krasnodar Clinical Centre for HIV Control in 1990 to 2015 were collected. This study was retrospective and non-interventional. All samples were initially analysed for diagnostic purposes within the Rostov-na-Donu-Cologne cooperation project (Ethics approval #246). Enrolment criteria were confirmed HIV infection and availability of one blood sample and provided written consent. Three hundred twenty-eight patients fulfilled these criteria. The final inclusion criterion for the study was availability of at least the reverse transcriptase (RT) and/or protease (PR) sequence from the blood sample. Three hundred three patients were included in the analyses.

Epidemiological data concerning reported transmission route, date of birth, and sex were collected at the treating sites. Data were anonymised for the study and stored in the Arevir database.

Viral RNA isolation and RT-PCR amplification for protease-reverse transcriptase (PRRT), reverse transcriptase (RT), and integrase (IN) were completed as previously described [20] with subtype-optimized primers. Next-Generation-Sequencing (MiSeq, Illumina) with a 15% cut-off was performed. Initial subtyping was done based on the RT (or PR region when the RT was not available) using the geno2pheno system (http://www.geno2pheno.org/) and Stanford HIVdb PROGRAM (HIVdb; https://hivdb.stanford.edu/hivdb/by-mutations/). Final subtyping of A1 or A-containing recombinant forms was based on phylogenetic analysis.

Four main datasets were used for the phylogeny: i) RUS: 222 RT sequences from our patients, initially predicted as A1 or one of the A-containing recombinants forms 01_AE, 02_AG, or 03_AE; ii) RUS-A6: 211 samples from the RUS dataset classified as A6 through our first phylogenetic analysis; iii) A6-POL-LA: 68 available A6-annotated *pol* sequences from the Los Alamos database (LA) (GenBank accession numbers in File S1); and iv) REF-LA: 40 subtype reference sequences from LA (Table S1) [21]. Two additional datasets were used for subtype prevalence comparison: v) RU-LA: 8355 LA HIV-1 sequences (any region) from Russia; and vi) FSU-LA: 12510 LA HIV-1 sequences (any region) from the former Soviet Union (FSU) countries (including the 8388 from RU-LA). An overview of all datasets is provided in Table S2.

LA searches were performed using the *search DB tool* (https://www.hiv.lanl.gov/components/sequence/HIV/search/search.html), or the *Geography Search Interface* (https://www.hiv.lanl.gov/components/sequence/HIV/geo/geo.comp) with “former USSR” or “Russian federation” as selected regions. Problematic sequences (classified as such in LA; https://www.hiv.lanl.gov/components/sequence/HIV/search/help.html#bad_seq) and entries without annotated subtypes were excluded.

Phylogenetic analyses were performed using the Mega 7 software [22]. Multiple alignments of DNA sequences were generated using the Muscle tool using UPGMB for clustering with a minimum diagonal length of 24 and a maximum of eight iterations [23]. These alignments were used to compute nucleotide pairwise distances and neighbour-joining (NJ) analysis, using the maximum composite method in both cases.

A6 consensus sequences were generated using MutExt software (E. Schülter, University of Cologne) and the Advanced Consensus Maker tool from Los Alamos (https://www.hiv.lanl.gov/content/sequence/CONSENSUS/AdvCon.html). High cut-offs of 70%–90% and lower cut-offs of 30%–50% were used. 

Resistance-associated-mutations (RAMs) and drug susceptibility profiles were analysed with the Stanford HIVdb [24]. Resistance levels of 1–2 were considered susceptible, levels of 3–4 as intermediate resistant, and 5 as fully resistant. Resistance levels 3–5 were considered clinically relevant. Drug class susceptibility was calculated as the highest level of resistance for any of the drugs included within the class.

We performed descriptive analyses for patients’ sex, year of birth, and transmission route as well as for viral subtypes, circulating recombinant forms (CRFs), RAMs, and drug susceptibility. We present genetic data as median pairwise distances with interquartile range values.

Statistical analysis of categorical variables was performed with the Fisher´s exact probability test using 2 × 2 contingency tables (http://vassarstats.net/tab2x2.html). All *p* values were two-tailed. Significant differences were considered as *p* values < 0.05.

## 3. Results

### 3.1. Baseline Characteristics

**Figure 1:**
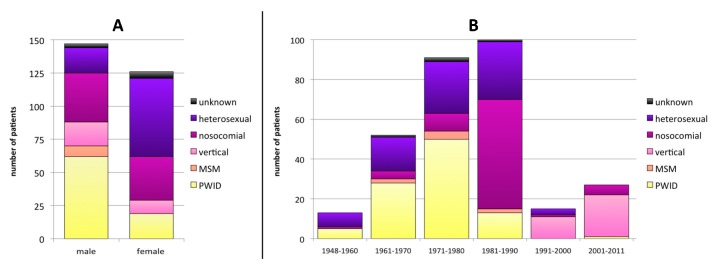
Transmission route distribution according to sex (**A**) and the year of birth (**B**). MSM: men having sex with men; PWID: persons who inject drugs.

Twenty-five samples could not be amplified in the RT and/or PR regions due to low viral load. Therefore 303/328 patients were included in the final analysis. One hundred fifty (49.5%) individuals were male, 126 (38.4%) female, and 27 (8.9%) unknown (not reported). In terms of the disclosed transmission routes, 97 (32.0%) patients were persons who inject drugs (PWID), 82 (27.1%) were heterosexuals, 76 (25.1%) were from late-1980´s nosocomial infections, 32 (10.6%) were vertical infections, 8 (2.6%) were men having sex with men (MSM), and for 8 (2.6%) the transmission route was not reported. Males were significantly more often PWID (42.2% of male infections; *p* < 0.001), whereas females were more likely to have been infected through heterosexual contact (46.8% of female infections; *p* < 0.001) (Figure 1A).

The year of birth was known for 298 patients: 65 (21.8%) were born in 1948–1970, 191 (64.1%) in 1971–1990, and 42 (14.1%) in 1991–2011 (Figure 1B). No clear changes in the sex distribution pattern based on the year of birth were detected (Table S3). Nosocomial infections were associated to a birth year between 1981 and 1990 (55.0% in this age group; *p* < 0.001). Infections in PWID were significantly more frequent in patients born in 1948–1980 (53.0% of all infections in patients in this age group) than in younger patients born in or after 1981 (9.9%; *p* < 0.001). Heterosexual transmission rate decreased with the patients´ age from 53.8% (birth 1948–1960) to 20.0% (1991–2011).

Therapy information was available for 249/303 patients, of which 121 were therapy-naïve (TN) at sampling time and 128 therapy-experienced (TE). All TE patients had received NRTIs. Twenty-two TE patients had undergone one zidovudine (AZT) monotherapy and 2 patients two AZT monotherapies. In addition, 65/128 (50.8%) patients had been treated with NNRTIs, 59/128 (46.1%) with PIs, and one (0.8%) with INSTIs.

### 3.2. Viral Subtype Distribution

**Figure 2:**
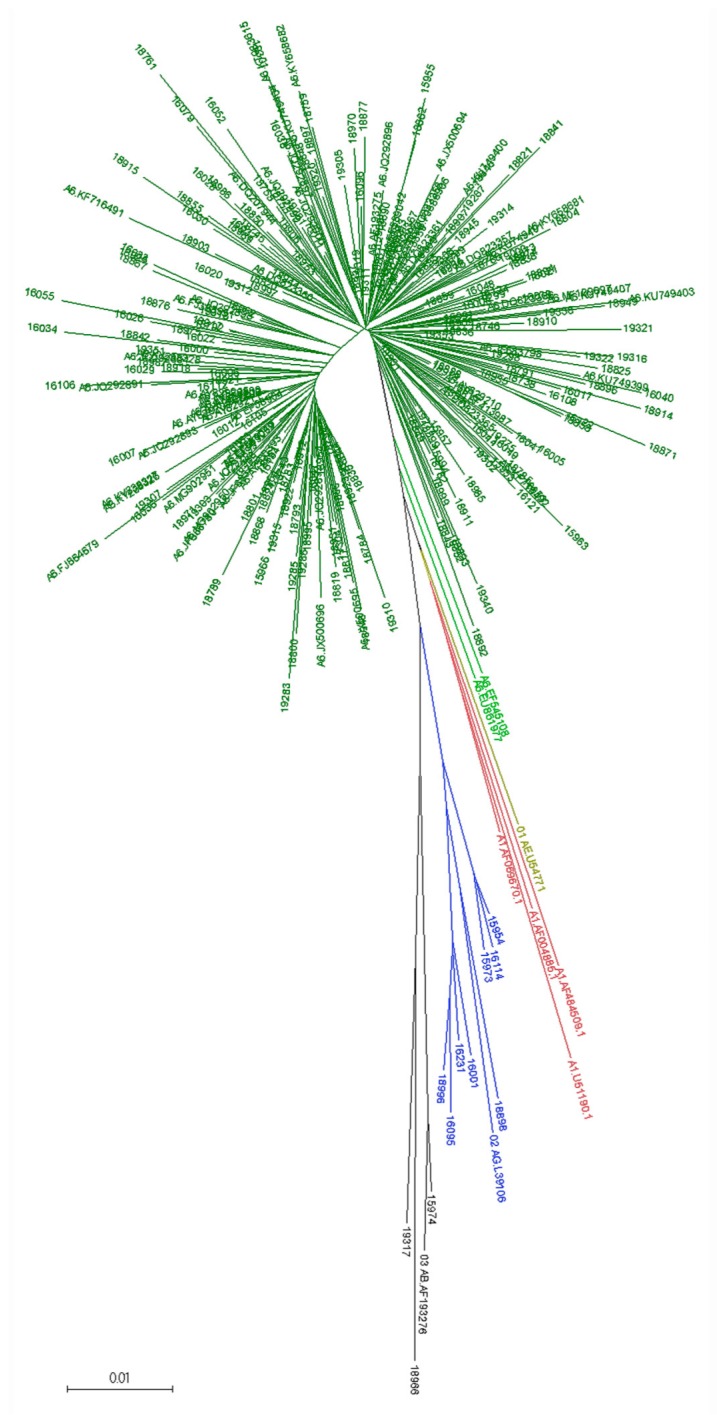
Neighbour-joining (NJ) tree for the identification of A6 samples. Samples EF545108 and EU861977 are highlighted in lime green.

Initial subtyping classified 222 RT samples predicted as subtype A or as subtype A-containing recombinant forms. They were subjected to phylogenetic analysis together with the A6-POL-LA dataset, and A1, 02_AG, 01_AE, and 03_AB reference sequences. The generated NJ tree showed a cluster comprising 211 RUS samples and 66/68 A6-POL-LA sequences; EF545108, EU861977 sequences located intermediate between A6, CRF01_AE, and A1 sequences (Figure 2). This analysis classified two initially A1-predicted sequences as 03_AB and one A1 as 02_AG. The 01_AE sample #16020 was reclassified as A6.

The RUS-A6 and A6-POL-LA datasets were used to generate ten different consensus sequences. The consensus sequence generated by MutExt using the high- and low-cut-offs of 70% and 50%, respectively, was selected as the reference consensus sequence (A6_pol_reference consensus sequence; nt. sequence in Supplementary Material, page 1). It showed the lowest maximal pairwise distance (0.034) as well as median distance (0.014; IQR 0.010–0.019) to any A6 sequence (Figure S1). EF545108 and EU861977 were the most distant sequences, with genetic distances to the A6_pol_reference consensus sequence of 0.031 and 0.034, respectively. These two sequences were still closer to A6 than to A1, as the minimal pairwise distances to any A1 reference sequence were 0.069 and 0.062, and the median distances to the four A1 references were 0.077 (0.073–0.083) and 0.070 (0.067–0.074), respectively. Subsequently, the A6_pol_reference consensus sequence was aligned to the REF-LA dataset (Figure S2). The A1 reference sequences were the closest genetically, with a minimal pairwise distance of 0.050 (Table S4). The maximal pairwise distance to our reference consensus sequence for a query sample to be classified as A6 was selected as 0.040. This cut-off significantly discriminated A6 samples in the RUS dataset (*p* < 0.001).

Applying the above criteria, the final subtype distribution in the 303 Southern Russia sequences was: 211 (69.6%) A6, 71 (23.4%) G, 10 (3.3%) B, 8 (2.6%) CRF 02_AG, and 3 (1.0%) CRF 03_AB (GenBank accession numbers MK029062-MK029336). Detailed information on subtypes and CRFs prevalence in the Southern Federal District, Russia (RU-LA dataset) and FSU countries (FSU-LA dataset) are listed in Table S5. Subtype G was significantly more prevalent in our Southern Russian dataset (*p* < 0.001) while the CRF63_02A was significantly more prevalent in the FSU-LA than in the RU-LA dataset (*p* < 0.001).

Heterogeneity analysis within the RUS-A6 dataset analysis showed a maximal genetic distance of 0.034, and a median of 0.013 (0.025–0.040). Subtype G isolates showed higher variability, with a maximal pair distance of 0.090 and an overall median distance of 0.042 (0.030–0.053).

**Figure 3:**
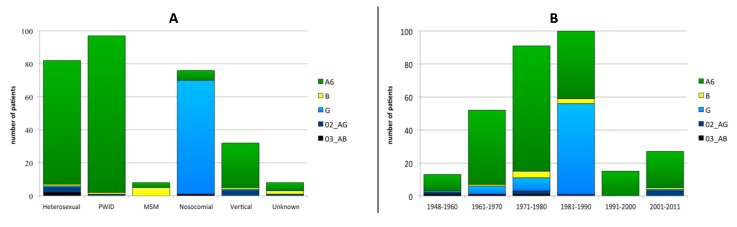
HIV-1 subtype distribution according to transmission route (**A**) and the year of birth (**B**) PWID: persons who inject drugs; MSM: men having sex with men.

There were no significant divergences in the subtype distribution between males and females. On the other hand, differences were found in subtype distribution depending on the transmission route (Figure 3A). Subtype G detection correlated with nosocomial infections (90.8%; *p* < 0.001) and subtype B infections significantly associated to MSM (62.5%; *p* < 0.001). The subtype distribution was independent of patients´ date of birth, with the exception of subtype G infections, which significantly correlated with a birth date between 1981 and 1990 (55.0%; *p* < 0.001; Figure 3B). This subtype was detected only in patients born before 1991 with only one exception: one vertical transmission in 2005 from a patient nosocomially infection in the late 1980s.

### 3.3. Resistance-Associated-Mutations (RAMs) and Drug-Susceptibility Profiles

Two hundred sixty-one PR, 277 RT, and 61 IN sequences were available and screened for RAMs (Table 1; Table S6). Two PI-, six NRTI-, and four NNRTI-RAMs were found to be significantly associated with therapy experience.

Detection of ≥2 NRTI-RAMs correlated with a history of ≥3 NRTIs (*p* < 0.001) and with a record of AZT monotherapy (*p* < 0.001). Detection of ≥1 NNRTI-RAMs or major PI-RAMs correlated with a history of ≥1 NRTIs or PIs, (*p* < 0.001 for both cases).

The prevalence of specific RAMs significantly correlated to viral subtype. RAMs I54AV_PR_, L90M_PR_, M41L_RT_, D67N_PR_, and T215F_PR_ were significantly more frequent in subtype G isolates. Conversely, A62V_PR_ and G190S_PR_ correlated with A6 infections. A62V_PR_ was detected in 60/191 (31.4%) of the A6 sequences. No significant difference in the prevalence between TN (29/76; 38.2%) and TE (22/75; 29.3%) patients was observed. On the other hand, G190S_PR_ was not detected in any TN patient but in 14/75 (18.7%) of the TE patients. Moreover, G190S_PR_ was observed in 16/30 (53.3%) of the efavirenz (EFV)-exposed patients, resulting in a significant association of this RAM in the context of A6 and EFV-exposure (*p* < 0.001).

Drug class susceptibility data are provided in Table 2. Transmitted drug resistance (TDR) was detected in TN patients: 8.8% of the isolates showed transmitted resistance to PIs and 6.4% to NRTIs. No TDR to NNRTIs or INSTIs was detected. In TE patients, 17.3% of the PR sequences were clinically resistant to PIs, 50.0% to NRTIs, 39.2% to NNRTIs, and 9.5% to INSTIs. Multiresistance was detected in 52 isolates, 40 corresponding to two-class and seven to three-class multiresistance.

## 4. Discussion

This study provides a comprehensive description of the Southern Russian HIV-1 epidemiology using samples obtained from 303 patients attending the major treatment centres in the Russian Southern Federal District. These data complement previous reports conducted in different Russian regions and with different epidemiological parameters [2,4,25]. The limited number of patients and the predominance of older patients in this study compared to others from West/Central Europe and North America (http://www.cdc.gov/hiv/library/reports/hiv-surveillance.html) is attributable to the high HIV unawareness and reduced accessibility to HIV care in Russia.

In Russia compared to western countries, HIV-1 epidemiology differs in several aspects. First, females are a vulnerable collective in Russia. Moreover, we show that the female proportion of HIV patients in medical care has not significantly decreased over the years in the Southern Federal District. In addition, PWID and heterosexual contacts are the main reported transmission modes in Russia [25], while in western countries, the impact of these two transmission modes is limited. In West/Central Europe and North America, MSM is the most affected group, both in cumulative incidence and new infections, and these infections correlate with subtype B [1]. In Eastern Europe and Central Asia, MSM represent only 6% of new infections, though they also correlate with this subtype [3,26]. Specifically, in Southern Russia less than 3% of the patients reported to be MSM and this transmission route also correlated with subtype B viruses. Yet, 25% of our samples with unknown transmission route were classified as subtype B, suggesting that they may indeed represent cases of withheld MSM transmission. Underrepresentation of MSM in epidemiological analyses is indeed expected as marked social discrimination complicates their enrolment in clinical studies and, upon inclusion, some of them may report a different transmission route, mostly heterosexual [2,3,26].

One of the major technical problems for Russian epidemiology analysis is the accurate subtyping of isolates. This is a key issue for sub-subtype A6, the most prevalent in FSU countries, but also for other CRFs such as 63_02A, which comprises 10.3% of the FSU sequences deposited in GenBank. These isolates cannot be identified by current bioinformatics subtyping tools. Therefore, our initial efforts were directed to develop a methodology to systematise its identification and subsequent annotation in Los Alamos and GenBank databases. The use of the A6_pol_reference consensus sequence allows for a clear discrimination between A6 and A1 samples based on the *pol* nucleotide sequence. Our data do not support EF545108 and EU861977 as reference sequences, in spite of their use in a number of previous studies [7,12,13,14,15], as they cluster between African A1 and A6 sequences, suggesting that they are actually ancestors of the presently circulating A6 sub-subtype [14]. The subsequent analysis of A6 prevalence in our data set showed a large predominance of this sub-subtype, similar to what is described for other regions in the country. The initial expansion of this sub-subtype took place in PWID networks [3], but now it has extended to all other collectives. The A6 identification methodology developed in this work has been transferred to the geno2pheno system team who are already working to extend the subtyping tool. In the near future, the use of free-access, web-based subtyping tool(s) will enable not only the effortless and straightforward identification of A6 isolates in routine diagnostics, but also the characterisation of their prevalence worldwide. Of note, 4% of the A6-annotated entries in the Los Alamos database are isolates from countries geographically close to the FSU such as Mongolia, Germany, and Turkey, but also from more distant countries like Cyprus, Spain, The Republic of Korea, U.S.A., and Australia (Table S7). We acknowledge the limitation that our analyses were based only on the *pol* region. This region was prioritized because it is the main genomic region analysed in routine diagnostics for subtyping as well as for drug resistance testing. Current experiments in our group are investigating other genomic regions such as the *IN* gene or the V3 loop of the gp120 subunit of the Env protein to further optimize and facilitate the detection of A6 isolates. In addition, the methodology developed in this work can also be extended to normalise the classification of other relevant subtypes/CRFs not yet detected through subtyping tools.

Exceptional within HIV Russian epidemiology is the large prevalence of subtype G in the Southern Federal District [3,15]. Subtype G strains are otherwise mostly reported from Nigeria and West African countries [27,28] (Table S8). They are rare in western countries, with the exceptions of Spain and Portugal, where they account for up to 12% and 30% of the infections, respectively [29,30,31,32]. In Russia, subtype G infections are unusual outside the region covered in this study [33]. The subtype G viruses from the Southern Federal District can be retraced to an episode of clonal infection of more than 250 children after exposure to non-sterile needles in 1988–1989 [3,19]. This event still shapes the current HIV landscape, with subtype G prevalence of 23% of the isolates detected in this study. Almost-full genome analyses of three isolates from the nosocomially-infected cohort showed genetic similarity to African G isolates and dissimilarity to eight G viruses from other Russian regions, which were related to G/CRF14_BG isolates from the Iberian zone [19]. Importantly and contrary to the African and South European cases where subtype G viruses are currently being actively transmitted among the population by heterosexual contacts, this study could not detect any subtype G sample outside this nosocomially infected group, suggesting that these viruses may not have entered other transmission networks yet.

Non-B subtypes show differences in disease progression (summarised in [34]) and present genetic signatures and polymorphisms in amino acid residues associated to resistance in subtype B. Subtypes C, F, G, or CRF02_AG show different susceptibility to specific antiretrovirals and may develop RAMs, which are not favoured in subtype B strains [35,36]. Our analysis detected two specific RAMs significantly associated to A6 viruses: A62V_RT_ and G190S_RT_. In subtype B, A62V_RT_ is a compensatory mutation associated with NRTI-class resistance. A62V_RT_ is observed in two unusual mutational patterns: the Q151M complex and the T69SSS insertion complex. The A62V_RT_ alone is rare in subtype B, TN-samples as it reduces the replication capacity of these variants to 50% compared to the wild type (wt) virus [37]. In A6 samples, A62V_RT_ is an endemic polymorphism with similarly high prevalence among TN and TE patients [9,38,39]. These prevalence trends strongly suggest that this mutation probably does not lead to a sharp reduction of replication capacity in the context of sub-subtype A6, though no experimental data to confirm this hypothesis are available yet. What is more, it opens the question of whether NRTI class-resistance patterns may be favoured in the A6 context. The limited size of our dataset hinders any conclusion in this direction. The substitution G190S_RT_ confers high level resistance against doravirine, efavirenz, and nevirapine and reduces etravirine and rilpivirine susceptibility to levels of still unknown clinical relevance [24]. In non-A6 isolates, K103N_RT_ or Y181C_RT_ are preferentially detected after efavirenz exposure [40,41,42,43] while G190S_RT_ is rarely observed, probably due to high costs in replication capacity (in the subtype B context, 20% compared to the wt) [41]. Conversely, G190S development in A6 isolates is significantly higher (up to 30% to 60%) after EFV and/or nevirapine (NVP)-exposure and is favoured over the K103N and Y181C resistance pathways [10,38,39,42,43,44]. These subtype differences may be partially attributable to the nucleotide exchanges required to achieve the amino acid substitution: While one transition and one transversion (GGA to TCA or AGY) are required in non-A6 isolates, only one transition is required (GGC to AGC) in the A6 genetic background [42]. The effect of G190S_RT_ in replication capacity of A6 isolates is yet to be analysed.

Single class- and multiresistance to all antiretroviral drug classes available in Russia were detected in the present study, threatening the long-term success of antiretroviral therapy programs. In South Russian therapy-experienced patients, resistance levels are comparable to those described for TE, viraemic patients in western countries [45,46,47]. Whether the prevalence of drug resistance in Russia is currently declining, as described for Western Europe and the U.S.A. [45,46] is still unknown. Our study included too few samples from recent years to drive any conclusion in this matter. Further studies including additional samples from the last decade are required to clarify this question. We also identified transmission of drug resistance. PI-, NRTI-, and even PI + NRTI-resistant strains were detected in TN patients. PI-TDR in South Russia, detected in 9% of the TN patients, seems to be higher than in other European countries [45,48,49,50], which may be a consequence of the extended use of older PIs such as atazanavir, nelfinavir, or saquinavir compared to western countries, where these drugs have been mostly replaced by darunavir.

In conclusion, this study enables the systematisation of A6 identification and confirms the predominance of sub-subtype A6 and subtype G in Southern Russia. Additionally, it shows that resistance is circulating in viruses from both TN and TE patients, with the presence of specific RAMs associated to individual subtypes. Efforts to increase the implementation of routine epidemiology and surveillance will further improve therapy efficacy in this region.

## Figures and Tables

**Table 1:** Resistance-associated-mutations (RAMs) detected in the protease (PR), reverse transcriptase (RT) and integrase (IN) sequences according to antiretroviral class.

RAMs	Patients				
	Total ^1^	TN ^2^	TE ^3^	No Data ^4^	*p* Value ^5^
**PI**	**261**	**102**	**110**	**49**	
D30N	1 (0.4%)	0 (0.0%)	1 (0.9%)	0 (0.0%)	≥0.05
M46IL	13 (5.0%)	2 (2.0%)	9 (8.2%)	2 (4.1%)	≥0.05
G48V	2 (0.8%)	2 (2.0%)	0 (0.0%)	0 (0.0%)	≥0.05
I50VL	4 (1.5%)	0 (0.0%)	3 (2.7%)	1 (2.0%)	≥0.05
I54AV	8 (3.1%)	1 (1.0%)	6 (5.5%)	1 (2.0%)	≥0.05
L76V	2 (0.8%)	1 (1.0%)	1 (0.9%)	0 (0.0%)	≥0.05
**V82AFMST**	10 (3.8%)	1 (1.0%)	**8 (7.3%)**	1 (2.0%)	**0,0363**
N88S	1 (0.4%)	0 (0.0%)	0 (0.0%)	1 (2.0%)	≥0.05
**L90M**	10 (3.8%)	1 (1.0%)	**8 (7.3%)**	1 (2.0%)	**0,0363**
**NRTIs**	**277**	**109**	**120**	**48**	
M41L	22 (7.9%)	1 (0.9%)	**19 (15.8%)**	2 (4.2%)	**<0.0001**
E44D	1 (0.4%)	0 (0.0%)	1 (0.8%)	0 (0.0%)	≥0.05
A62V	61 (22.0%)	30 (27.5%)	22 (18.3%)	9 (18.8%)	≥0.05
D67G	3 (1.1%)	1 (0.9%)	2 (1.7%)	0 (0.0%)	≥0.05
**D67N**	17 (6.1%)	1 (0.9%)	**13 (10.8%)**	3 (6.3%)	**0.0016**
K65DEN	4 (1.4%)	1 (0.9%)	3 (2.5%)	0 (0.0%)	≥0.05
T69DN	3 (1.1%)	0 (0.0%)	3 (2.5%)	0 (0.0%)	≥0.05
T69G_SG	1 (0.4%)	0 (0.0%)	1 (0.8%)	0 (0.0%)	≥0.05
**K70ER**	14 (5.1%)	1 (0.9%)	**11 (9.2%)**	2 (4.2%)	**0.0058**
L74IV	9 (3.2%)	0 (0.0%)	6 (5.0%)	3 (6.3%)	≥0.05
V75AIM	5 (1.8%)	0 (0.0%)	5 (4.2%)	0 (0.0%)	≥0.05
Y115F	2 (0.7%)	0 (0.0%)	1 (0.8%)	1 (2.1%)	≥0.05
**M184IV**	60 (21.7%)	5 (4.6%)	**44 (36.7%)**	11 (22.9%)	**<0.0001**
**L210W**	8 (2.9%)	0 (0.0%)	**8 (6.7%)**	0 (0.0%)	**0.0073**
**T215CFILNSY**	38 (13.7%)	1 (0.9%)	**33 (27.5%)**	4 (8.3%)	**<0.0001**
**K219ENQ**	16 (5.8%)	1 (0.9%)	**12 (10.0%)**	3 (6.3%)	**0.0030**
**NNRTI**	**277**	**109**	**120**	**48**	
A98G	2 (0.7%)	0 (0.0%)	2 (1.7%)	0 (0.0%)	≥0.05
**K101EHPQ**	11 (4.0%)	0 (0.0%)	**9 (7.5%)**	2 (4.2%)	**0.0036**
L100F	2 (0.7%)	0 (0.0%)	2 (1.7%)	0 (0.0%)	≥0.05
**K103NS**	28 (10.1%)	0 (0.0%)	**24 (20.0%)**	4 (8.3%)	**<0.0001**
V106A	1 (0.4%)	0 (0.0%)	1 (0.8%)	0 (0.0%)	≥0.05
V108I	6 (2.2%)	0 (0.0%)	3 (2.5%)	3 (6.3%)	≥0.05
E138AGHKQR	20 (7.2%)	5 (4.6%)	13 (10.8%)	2 (4.2%)	≥0.05
V179DE	6 (2.2%)	2 (1.8%)	4 (3.3%)	0 (0.0%)	≥0.05
**Y181CFIV**	13 (4.7%)	0 (0.0%)	**11 (9.2%)**	2 (4.2%)	**0.0009**
Y188LS	4 (1.4%)	1 (0.9%)	3 (2.5%)	0 (0.0%)	≥0.05
**G190AS**	21 (7.6%)	0 (0.0%)	**18 (15.0%)**	3 (6.3%)	**<0.0001**
H221Y	2 (0.7%)	0 (0.0%)	2 (1.7%)	0 (0.0%)	≥0.05
P225H	4 (1.4%)	0 (0.0%)	3 (2.5%)	1 (2.1%)	≥0.05
F227L	1 (0.4%)	0 (0.0%)	1 (0.8%)	0 (0.0%)	≥0.05
M230L	1 (0.4%)	0 (0.0%)	1 (0.8%)	0 (0.0%)	≥0.05
K238T	1 (0.4%)	0 (0.0%)	0 (0.0%)	1 (2.1%)	≥0.05
Y318F	1 (0.4%)	0 (0.0%)	1 (0.8%)	0 (0.0%)	≥0.05
**INSTI**	**61**	**19**	**21**	**21**	
T6TK	1 (1.6%)	0 (0.0%)	1 (4.8%)	0 (0.0%)	≥0.05
G140A	1 (1.6%)	0 (0.0%)	1 (4.8%)	0 (0.0%)	≥0.05
Q148R	1 (1.6%)	0 (0.0%)	1 (4.8%)	0 (0.0%)	≥0.05
N155H	1 (1.6%)	0 (0.0%)	1 (4.8%)	0 (0.0%)	≥0.05

^1^ All available sequences; ^2^ samples from therapy-naive (TN) patients; ^3^ samples from therapy-experienced (TE) patients; ^4^ samples from patients whose therapy records were not available; ^5^ calculated considering only TN versus TE samples. T69G_SG indicates an insertion of S in residue number 69. Statistically significant differences between TN and TE are highlighted in bold.

**Table 2:** Number of available sequences and drug susceptibility.

		Patients			
		Total ^1^	TN ^2^	TE ^3^	No Data ^4^
**Available PR Sequences**		**261**	**102**	**110**	**49**
PI	IR	11 (4.2%)	6 (5.9%)	5 (4.5%)	0 (0.0%)
	FR	21 (8.0%)	3 (2.9%)	14 (12.7%)	4 (8.2%)
	CR-resistance	32 (12.3%)	9 (8.8%)	19 (17.3%)	4 (8.2%)
**Available RT Sequences**		**277**	**109**	**120**	**48**
NRTI	IR	9 (3.2%)	2 (1.8%)	6 (5.0%)	1 (2.1%)
	FR	72 (26.0%)	5 (4.6%)	54 (45.0%)	13 (27.1%)
	CR-resistance	81 (29.2%)	7 (6.4%)	60 (50.0%)	14 (29.2%)
NNRTI	IR	2 (0.7%)	0 (0.0%)	1 (0.8%)	1 (2.1%)
	FR	54 (19.5%)	0 (0.0%)	46 (38.3%)	8 (16.7%)
	CR-resistance	56 (20.2%)	0 (0.0%)	47 (39.2%)	9 (18.8%)
NRTI + NNRTI	IR	3 (1.1%)	0 (0.0%)	2 (1.7%)	1 (2.1%)
	FR	30 (10.83%)	0 (0.0%)	26 (21.7%)	4 (8.3%)
	CR-resistance	33 (11.9%)	0 (0.0%)	28 (23.3%)	5 (10.4%)
**Available PR + RT Sequences**		**260**	**102**	**109**	**49**
PI + NRTI	IR	1 (0.4%)	1 (1.0%)	0 (0.0%)	0 (0.0%)
	FR	9 (3.5%)	2 (2.0%)	5 (4.6%)	2 (4.1%)
	CR-resistance	10 (3.8%)	3 (2.9%)	5 (4.6%)	2 (4.1%)
PI + NNRTI	IR	1 (0.4%)	0 (0.0%)	1 (0.9%)	0 (0.0%)
	FR	1 (0.4%)	0 (0.0%)	1 (0.9%)	0 (0.0%)
	CR-resistance	2 (0.8%)	0 (0.0%)	2 (1.8%)	0 (0.0%)
PI + NRTI + NNRTI	IR	0 (0.0%)	0 (0.0%)	0 (0.0%)	0 (0.0%)
	FR	5 (1.9%)	0 (0.0%)	3 (2.8%)	2 (4.1%)
	CR-resistance	5 (1.9%)	0 (0.0%)	3 (2.8%)	2 (4.1%)
**Available IN Sequences**		**61**	**19**	**21**	**21**
INSTI	IR	0 (0.0%)	0 (0.0%)	0 (0.0%)	0 (0.0%)
	FR	2 (3.3%)	0 (0.0%)	2 (9.5%)	2 (4.1%)
	CR-resistance	3 (3.3%)	0 (0.0%)	2 (9.5%)	2 (4.1%)
**Available RT + IN Sequences**		**52**	**18**	**17**	**17**
NRTI + NNRTI + INSTI	IR	0 (0.0%)	0 (0.0%)	0 (0.0%)	0 (0.0%)
	FR	2 (3.8%)	0 (0.0%)	2 (11.8%)	0 (0.0%)
	CR-resistance	2 (3.8%)	0 (0.0%)	2 (11.8%)	0 (0.0%)

^1^ All available sequences; ^2^ samples from therapy-naive patients; ^3^ samples from therapy-experienced patients; ^4^ samples from patients whose therapy records were not available; IR: intermediate resistance; FR: fully-resistant; CR-resistance: clinically relevant resistance, calculated as the addition of IR and FR.

## Supplementary Materials

The following are available online at https://www.mdpi.com/1999-4915/12/4/475/s1, Figure S1: Distribution of the pairwise genetic distances between the A6_pol_reference consensus and the A6 samples, Figure S2: NJ tree of A6_pol_reference consensus and HIV-1 subtype reference sequences, Table S1: GenBank accession numbers for the reference sequences, Table S2: Overview of the datasets used in this study, Table S3: Sex distribution based on the year of birth, Table S4: Pairwise distances between A6_pol_reference consensus and the REF-LA dataset, Table S5: HIV-1 subtype and CRF prevalence in GenBank and our Southern Russia samples, Table S6: Number of RAMs per sequence according drug class, Table S7: Country of origin of HIV-1 sequences (any genomic region) annotated as subclade A6 in the Los Alamos database, Table S8: Country of origin of HIV-1 sequences (any genomic region) annotated as subtype G in the Los Alamos database. File S1: GenBank accession numbers of sequences included in A6-POL-LA dataset.
