# Supplementary material for: HIV-1 Sub-Subtype A6: Settings for Normalised Identification and Molecular Epidemiology in the Southern Federal District, Russia
Source: Viruses. 2020 Apr 22;12(4):475. doi: 10.3390/v12040475 (PMC7232409; doi:10.3390/v12040475)
Supplement: Supplementary file 1 [file viruses-12-00475-s001.zip › viruses-764837-supplementary3/supplementary material/Fig S1.docx]

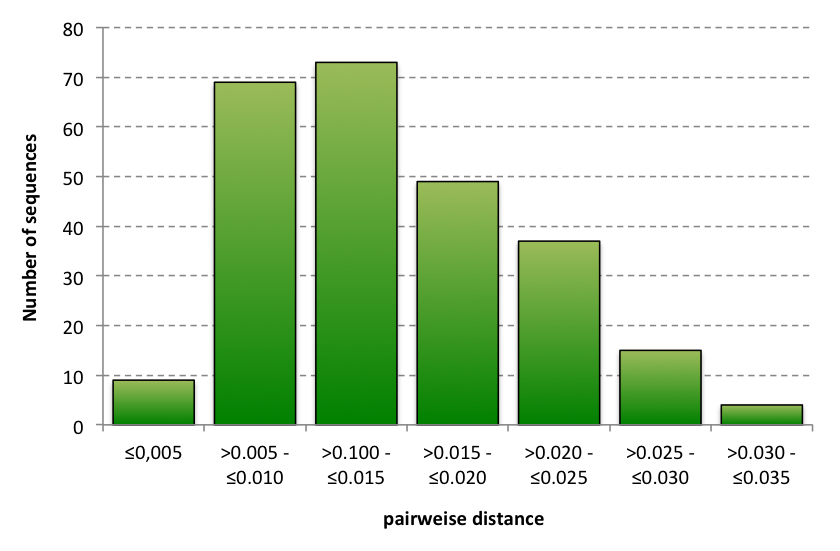


**Supplementary Fig. S1: distribution of the pairwise genetic distance between A6_pol_reference consensus and the A6 samples**
