# Supplementary material for: HIV-1 Sub-Subtype A6: Settings for Normalised Identification and Molecular Epidemiology in the Southern Federal District, Russia
Source: Viruses. 2020 Apr 22;12(4):475. doi: 10.3390/v12040475 (PMC7232409; doi:10.3390/v12040475)
Supplement: Supplementary file 1 [file viruses-12-00475-s001.zip › viruses-764837-supplementary3/supplementary material/File S1.docx]

**GenBank accession numbers of sequences included in A6-POL-LA dataset**

AF193275, AF413987, AY500393, AY829203, AY829205, AY829206, AY829208, AY829209, AY829210, AY829211, AY829212, DQ207944, DQ823356, DQ823357, DQ823358, DQ823359, DQ823360, DQ823361, DQ823365, DQ823366, DQ823367, EF589039, EF589040, EF589041, EF589042, EF589043, EF589044, FJ388892, FJ388906, FJ388950, FJ388951, FJ864679, JF683763, JF683780, JF683798, JQ292891, JQ292892, JQ292893, JQ292894, JQ292895, JQ292896, JQ292897, JQ292898, JQ292899, JQ292900, JX500694, JX500695, JX500696, KF716491, KF716492, KT983615, KU749399, KU749400, KU749401, KU749403, KU749404, KU749405, KU749407, KY238326, KY238327, KY238328, KY658681, KY658682, MF109697, MG902950, MG902951, EF545108, EU861977

**A6_pol_reference consensus sequence**

CCTCAAATCACTCTTTGGCAACGACCCCTTGTCACAATAAAAATAGGAGGACAGCTAAAAGAAGCTCTATTAGATACAGGAGCAGATGATACAGTATTAGAAGAMATAAATTTGCCAGGAAAATGGAAACCAAAAATGATAGGGGGAATTGGRGGTTTTATYAAAGTAARACAGTATGATCAGATAMTTATAGAAATTTGTGGAAAAAAGGCTATAGGTWCAGTATTAGTAGGRCCTACCCCTGTCAACATAATTGGAAGAAATATGTTGACTCARCTTGGTTGTACTTTAAATTTTCCAATAAGTCCTATTGAAACTGTACCAGTAAMATTAAAGCCAGGAATGGATGGCCCAAAGGTTAAACAATGGCCATTAACAGAAGAAAAAATAAAAGCATTAACAGAMATTTGTAAGGARATGGAAAAGGAAGGAAAAATTTCAAAAATTGGGCCTGAAAATCCATACAATACTCCARTATTTGCTATAAAGAAAAARGACAGCACTAAGTGGAGGAAATTAGTAGATTTCAGGGARCTYAATAAAARAAMTCAGGACTTTTGGGAAGTTCAATTAGGAATACCCCATCCAGCGGGTTTAAAAAARAAAAAATCAGTAACAGTACTWGATGTGGGGGATGCATATTTTTCAGTTCCYTTAGATGAAARCTTCAGAAAGTATACTGCATTCACTATACCAAGTATAAACAATGAGAMACCAGGGRTCAGRTATCARTACAATGTACTTCCACAGGGATGGAAAGGATCACCAACAATATTCCAGRGTAGCATGACAAAAATCTTAGAGCCCTTTAGATYAAAAAATCCAGAAATAGTTATCTATCAATACATGGATGACTTGTATGTAGGCTCTGATTTAGAAATAGGGCAACATAGARCAAAAATAGARGAGTTAAGARCTCATCTATTGAGCTGGGGATTTACTACACCAGACAAAAAGCATCARAAAGAACCTCCATTTCTTTGGATGGGRTATGAACTCCATCCTGACAAATGGACAGTCCAGCCTATARWGCTRCCARATAAAGACAGCTGGACTGTCAATGATATACAGAAATTAGTGGGAAAACTAAATTGGGCAAGTCARATTTATCCAGGGATTAAAGTAAAGCAATTGTGTAAACTCCTCAGGGGARCCAAAGCACTGACAGATATAGTGACACTGACTGAGGAAGCAGAATTAGAATTGGCAGAGAACAGRGAGATTCTAAAAGAACCTGTGCATGGRGTATATTATGACCCATCAAAAGATTTAGTAGCAGAAATACAGAAACAAGGACAAGACGTAGGAGCAGAGACTTTCTC
