# Supplementary material for: HIV-1 Sub-Subtype A6: Settings for Normalised Identification and Molecular Epidemiology in the Southern Federal District, Russia
Source: Viruses. 2020 Apr 22;12(4):475. doi: 10.3390/v12040475 (PMC7232409; doi:10.3390/v12040475)
Supplement: Supplementary file 1 [file viruses-12-00475-s001.zip › viruses-764837-supplementary3/supplementary material/Table S1.docx]

| Subtype | GenBank accession number |
| --- | --- |
| A1 | AF004885 |
| A1 | AF069670 |
| A1 | U51190 |
| A1 | AF484509 |
| A2 | AF286238 |
| A2 | AF286237 |
| B | K03455 |
| B | AY173951 |
| B | AY423387 |
| B | AY331295 |
| C | U46016 |
| C | U52953 |
| C | AF067155 |
| C | AY772699 |
| D | K03454 |
| D | U88824 |
| D | AY371157 |
| D | AY253311 |
| F1 | AF005494 |
| F1 | AF077336 |
| F1 | AF075703 |
| F1 | AJ249238 |
| F2 | AJ249236 |
| F2 | AJ249237 |
| F2 | AY371158 |
| F2 | AF377956 |
| G | AF061642 |
| G | AF061640 |
| G | AF084936 |
| G | U88826 |
| H | AF005496 |
| H | AF190127 |
| H | AF190128 |
| J | AF082394 |
| J | AF082395 |
| K | AJ249235 |
| K | AJ249239 |
| CRF 01_AE | U54771 |
| CRF 02_AG | L39106 |
| CRF 03_AB | AF193276 |

**Supplementary Table S1: GenBank accession numbers for the reference sequences (REF-LA dataset)**
