# Supplementary material for: HIV-1 Sub-Subtype A6: Settings for Normalised Identification and Molecular Epidemiology in the Southern Federal District, Russia
Source: Viruses. 2020 Apr 22;12(4):475. doi: 10.3390/v12040475 (PMC7232409; doi:10.3390/v12040475)
Supplement: Supplementary file 1 [file viruses-12-00475-s001.zip › viruses-764837-supplementary3/supplementary material/Table S2.docx]

| **Dataset name** | **No. of sequences** | **Samples origin** | **genomic region** | **HIV-1 subtype** |
| --- | --- | --- | --- | --- |
| RUS | 222 | patients from Southern Russia | p51 (RT) | A1, CRF01_AE, CRF02_AG, CRF03_AE* |
| RUS-A6 | 211 | patients from Southern Russia | p51 (RT) | A6 |
| A6-POL-LA | 68 | LA (FSU countries) | p51 (RT) | A6 |
| REF-LA | 37 | LA (worldwide) | full genome | all |
| FSU-LA | 12510 | LA (FSU countries) | any | all |
| RU-LA | 8388 | LA (Russia) | any | all |
| A6-WORLD-LA | 9242 | LA (worldwide) | any | A6 |

**Supplementary Table S2: Overview of the datasets used in this study**

LA: Los Alamos database; FSU: former Soviet Union; *initial subtyping before phylogenetic analysis
