# Supplementary material for: HIV-1 Sub-Subtype A6: Settings for Normalised Identification and Molecular Epidemiology in the Southern Federal District, Russia
Source: Viruses. 2020 Apr 22;12(4):475. doi: 10.3390/v12040475 (PMC7232409; doi:10.3390/v12040475)
Supplement: Supplementary file 1 [file viruses-12-00475-s001.zip › viruses-764837-supplementary3/supplementary material/Table S3.docx]

| **year of birth** | **male** | **female** |
| --- | --- | --- |
| 1948-1960 | 3 (27.3%) | 8 (72.7%) |
| 1961-1970 | 25 (53.2%) | 22 (46.8%) |
| 1971-1980 | 49 (61.3%) | 31 (38.8%) |
| 1981-1990 | 49 (51.0%) | 47 (49.0%) |
| 1991-2000 | 7 (58.3%) | 5 (41.7%) |
| 2001-2011 | 16 (64.0%) | 9 (36.0%) |
| **Total** | **149** | **122** |

**Supplementary Table S3: Sex distribution based on the year of birth**
