# Supplementary material for: HIV-1 Sub-Subtype A6: Settings for Normalised Identification and Molecular Epidemiology in the Southern Federal District, Russia
Source: Viruses. 2020 Apr 22;12(4):475. doi: 10.3390/v12040475 (PMC7232409; doi:10.3390/v12040475)
Supplement: Supplementary file 1 [file viruses-12-00475-s001.zip › viruses-764837-supplementary3/supplementary material/Table S4.docx]

| **Subtype** | **median distance (IQR)** | **max. distance** | **min. distance** |
| --- | --- | --- | --- |
| A1 | 0.057 (0.053-0.059) | 0.066 | 0.050 |
| A2 | 0.081 (0.080-0.081) | 0.082 | 0.079 |
| B | 0.095 (0.093-0.097) | 0.098 | 0.091 |
| C | 0.095 (0.095-0.096) | 0.098 | 0.093 |
| D | 0.100 (0.098-0.102) | 0.102 | 0.098 |
| F1 | 0.092 (0.088-0.095) | 0.098 | 0.087 |
| F2 | 0.094 (0.093-0.094) | 0.096 | 0.092 |
| G | 0.098 (0.092-0.104) | 0.105 | 0.092 |
| H | 0.099 (0.095-0.105) | 0.109 | 0.088 |
| J | 0.114 (0.114-0.114) | 0.114 | 0.114 |
| K | 0.079 (0.078-0.088) | 0.096 | 0.079 |

**Supplementary Table S4: Pairwise distances between A6_pol_reference consensus and the REF-LA dataset**
