# Supplementary material for: HIV-1 Sub-Subtype A6: Settings for Normalised Identification and Molecular Epidemiology in the Southern Federal District, Russia
Source: Viruses. 2020 Apr 22;12(4):475. doi: 10.3390/v12040475 (PMC7232409; doi:10.3390/v12040475)
Supplement: Supplementary file 1 [file viruses-12-00475-s001.zip › viruses-764837-supplementary3/supplementary material/Table S5.docx]

| **Subtype^a^** | **FSU-LA dataset** | | **RU-LA dataset** | | **Southern Russia** |
| --- | --- | --- | --- | --- | --- |
|  | **any region (n=12510)** | **p51^b^ (n=164)** | **any region (n=8388)** | **P51 (n=102)^c^** | **PR/RT (n=303)** |
| A | 828 (6.6%) | 0 (0.0%) | 527 (6.3%) | 0 (0.0%) | 0 (0.0%) |
| A1 | 19 (0.2%) | 0 (0.0%) | 15 (0.2%) | 0 (0.0%) | 0 (0.0%) |
| A6 | 7962 (63.6%) | 110 (67.1%) | 5690 (67.8%) | 63 (61.8%) | 211 (69.6%) |
| B | 767 (6.1%) | 11 (6.7%) | 579 (6.9%) | 7 (6.9%) | 10 (3.3%) |
| C | 72 (0.6%) | 1 (0.6%) | 57 (0.7%) | 0 (0.0%) | 0 (0.0%) |
| D | 5 (0.0%) | 0 (0.0%) | 2 (0.0%) | 0 (0.0%) | 0 (0.0%) |
| F | 2 (0.0%) | 0 (0.0%) | 2 (0.0%) | 0 (0.0%) | 0 (0.0%) |
| F1 | 14 (0.1%) | 0 (0.0%) | 14 (0.2%) | 1 (1.0%) | 0 (0.0%) |
| F2 | 1 (0.0%) | 0 (0.0%) | 1 (0.0%) | 0 (0.0%) | 0 (0.0%) |
| **G** | **395 (3.2%)** | **2 (1.2%)** | **388 (4.6%)** | **4 (3.9%)** | **71 (23.4%)** |
| H | 5 (0.0%) | 0 (0.0%) | 5 (0.1%) | 0 (0.0%) | 0 (0.0%) |
| CRF01_AE | 14 (0.1%) | 0 (0.0%) | 14 (0.2%) | 0 (0.0%) | 0 (0.0%) |
| CRF02_AG | 421 (3.4%) | 0 (0.0%) | 31 (0.4%) | 0 (0.0%) | 8 (2.6%) |
| CRF03_AB | 295 (2.4%) | 5 (3.0%) | 227 (2.7%) | 2 (2.0%) | 3 (1.0%) |
| CRF06_cpx | 21 (0.2%) | 0 (0.0%) | 0 (0.0%) | 1 (1.0%) | 0 (0.0%) |
| CRF07_BC | 2 (0.0%) | 0 (0.0%) | 0 (0.0%) | 0 (0.0%) | 0 (0.0%) |
| CRF11_cpx | 2 (0.0%) | 0 (0.0%) | 2 (0.0%) | 0 (0.0%) | 0 (0.0%) |
| CRF18_cpx | 1 (0.0%) | 0 (0.0%) | 0 (0.0%) | 0 (0.0%) | 0 (0.0%) |
| CFR30_02A6 | 258 (2.1%) | 0 (0.0%) | 0 (0.0%) | 5 (4.9%) | 0 (0.0%) |
| CRF32_06A6 | 7 (0.1%) | 1 (0.6%) | 7 (0.1%) | 0 (0.0%) | 0 (0.0%) |
| CRF35_AD | 1 (0.0%) | 0 (0.0%) | 0 (0.0%) | 0 (0.0%) | 0 (0.0%) |
| CRF43_02G | 1 (0.0%) | 0 (0.0%) | 0 (0.0%) | 0 (0.0%) | 0 (0.0%) |
| **CRF63_02A** | **1294 (10.3%)** | **29 (17.7%)** | **740 (8.8%)** | **11 (10.8%)** | **0 (0.0%)** |
| 01G | 1 (0.0%) | 0 (0.0%) | 0 (0.0%) | 0 (0.0%) | 0 (0.0%) |
| O263 | 8 (0.1%) | 0 (0.0%) | 2 (0.0%) | 0 (0.0%) | 0 (0.0%) |
| 02A1 | 1 (0.0%) | 0 (0.0%) | 1 (0.0%) | 1 (1.0%) | 0 (0.0%) |
| 02B | 1 (0.0%) | 0 (0.0%) | 0 (0.0%) | 0 (0.0%) | 0 (0.0%) |
| 02F2 | 1 (0.0%) | 0 (0.0%) | 1 (0.0%) | 0 (0.0%) | 0 (0.0%) |
| 03A6 | 1 (0.0%) | 0 (0.0%) | 0 (0.0%) | 0 (0.0%) | 0 (0.0%) |
| 63A6 | 28 (0.2%) | 0 (0.0%) | 16 (0.2%) | 0 (0.0%) | 0 (0.0%) |
| A1A6 | 2 (0.0%) | 0 (0.0%) | 0 (0.0%) | 0 (0.0%) | 0 (0.0%) |
| A1B | 5 (0.0%) | 0 (0.0%) | 1 (0.0%) | 0 (0.0%) | 0 (0.0%) |
| A1BG | 2 (0.0%) | 0 (0.0%) | 2 (0.0%) | 2 (2.0%) | 0 (0.0%) |
| A6B | 40 (0.3%) | 0 (0.0%) | 35 (0.4%) | 0 (0.0%) | 0 (0.0%) |
| A6G | 3 (0.0%) | 0 (0.0%) | 3 (0.0%) | 0 (0.0%) | 0 (0.0%) |
| AB | 15 (0.1%) | 0 (0.0%) | 14 (0.2%) | 0 (0.0%) | 0 (0.0%) |
| AD | 3 (0.0%) | 0 (0.0%) | 0 (0.0%) | 0 (0.0%) | 0 (0.0%) |
| BG | 5 (0.0%) | 5 (3.0%) | 5 (0.1%) | 5 (4.9%) | 0 (0.0%) |
| DG | 5 (0.0%) | 0 (0.0%) | 5 (0.1%) | 0 (0.0%) | 0 (0.0%) |
| FU | 1 (0.0%) | 0 (0.0%) | 1 (0.0%) | 0 (0.0%) | 0 (0.0%) |
| U | 1 (0.0%) | 0 (0.0%) | 1 (0.0%) | 0 (0.0%) | 0 (0.0%) |

**Supplementary Table S5: HIV-1 subtype and CRF prevalence in GenBank and our Southern Russia samples**

^a^annotated (for LA samples) or determined (for the 303 Southern Russia samples) subtype or CRF; ^b^all p51-annotated samples included in the FSU-LA dataset; ^c^all p51-annotated samples included in the RU-LA dataset. Statistically significant differences between our results and LA datasets are highlighted in bold.
