# Supplementary material for: HIV-1 Sub-Subtype A6: Settings for Normalised Identification and Molecular Epidemiology in the Southern Federal District, Russia
Source: Viruses. 2020 Apr 22;12(4):475. doi: 10.3390/v12040475 (PMC7232409; doi:10.3390/v12040475)
Supplement: Supplementary file 1 [file viruses-12-00475-s001.zip › viruses-764837-supplementary3/supplementary material/Table S6.docx]

| **RAMs per sample** | **Number of sequences with RAMs** | | | |
| --- | --- | --- | --- | --- |
|  | **PI** | **NRTI** | **NNRTI** | **INI** |
| 0 RAMs | 237 (90.8%) | 148 (53.4%) | 200 (72.2%) | 59 (96.7%) |
| 1 RAM | 10 (3.8%) | 77 (27.8%) | 49 (17.7%) | 1 (1.6%) |
| 2 RAMs | 7 (2.7%) | 21 (7.6%) | 17 (6.1%) | 0 (0.0%) |
| 3 RAMs | 2 (0.8%) | 10 (3.6%) | 8 (2.9%) | 1 (1.6%) |
| 4 RAMs | 4 (1.5%) | 9 (3.2%) | 1 (0.4%) | 0 (0.0%) |
| 5 RAMs | 1 (0.4%) | 6 (2.2%) | 2 (0.7%) | 0 (0.0%) |
| 6 RAMs | 0 (0.0%) | 3 (1.1%) | 0 (0.0%) | 0 (0.0%) |
| 7 RAMs | 0 (0.0%) | 3 (1.1%) | 0 (0.0%) | 0 (0.0%) |

**Supplementary Table S6: Number of RAMs per sequence according drug class**
