# Supplementary material for: HIV-1 Sub-Subtype A6: Settings for Normalised Identification and Molecular Epidemiology in the Southern Federal District, Russia
Source: Viruses. 2020 Apr 22;12(4):475. doi: 10.3390/v12040475 (PMC7232409; doi:10.3390/v12040475)
Supplement: Supplementary file 1 [file viruses-12-00475-s001.zip › viruses-764837-supplementary3/supplementary material/Table S7.docx]

| **FSU country** | | **Non-FSU country** | | | |
| --- | --- | --- | --- | --- | --- |
| Armenia | 186 (2.0%) | Australia | 6 (0.1%) | Portugal | 2 (0.0%) |
| Azerbaijan | 37 (0.4%) | Austria | 2 (0.0%) | Republic of Korea | 4 (0.0%) |
| Belarus | 367 (4%) | Belgium | 2 (0.0%) | Spain | 44 (0.5%) |
| Bulgaria | 4 (0%) | China | 20 (0.2%) | Sweden | 8 (0.1%) |
| Czech Republic | 126 (1.4%) | Cyprus | 35 (0.4%) | Switzerland | 2 (0.0%) |
| Estonia | 61 (0.7%) | Democratic Republic of the Congo | 1 (0.0%) | Turkey | 37 (0.4%) |
| Georgia | 30 (0.3%) | Denmark | 3 (0.0%) | U.S.A. | 16 (0.2%) |
| Hungary | 5 (0.1%) | Finland | 3 (0.0%) | UK | 32 (0.3%) |
| Kazakhstan | 251 (2.7%) | France | 10 (0.1%) | Unknown | 1 (0.0%) |
| Kyrgyzstan | 155 (1.7%) | Germany | 110 (1.2%) |  | |
| Latvia | 612 (6.6%) | Greece | 1 (0.0%) |  |  |
| Lithuania | 18 (0.2%) | India | 2 (0.0%) |  |  |
| Poland | 51 (0.6%) | Italy | 6 (0.1%) |  |  |
| Republic of Moldova | 12 (0.1%) | Japan | 7 (0.1%) |  |  |
| Russian Federation | 5690 (61.6%) | Kuwait | 1 (0.0%) |  |  |
| Slovenia | 21 (0.2%) | Mongolia | 10 (0.1%) |  |  |
| Tajikistan | 276 (3%) | Netherlands | 1 (0.0%) |  |  |
| Ukraine | 682 (7.4%) | Norway | 2 (0.0%) |  |  |
| Uzbekistan | 288 (3.1%) | Philippines | 2 (0.0%) |  |  |
| **Total FSU: 8872 (96%)** | | **Total non-FSU: 370 (4.0%)** | | | |

**Supplementary Table S7: Country of origin of HIV-1 sequences (any genomic region) annotated as subclade A6 in Los Alamos database**

FSU: former Soviet Union country
