# Supplementary material for: HIV-1 Sub-Subtype A6: Settings for Normalised Identification and Molecular Epidemiology in the Southern Federal District, Russia
Source: Viruses. 2020 Apr 22;12(4):475. doi: 10.3390/v12040475 (PMC7232409; doi:10.3390/v12040475)
Supplement: Supplementary file 1 [file viruses-12-00475-s001.zip › viruses-764837-supplementary3/supplementary material/Table S8.docx]

| **FSU Country** | | **Non-FSU Country** | | | |
| --- | --- | --- | --- | --- | --- |
| Belarus | 2 (0.0%) | Afghanistan | 3 (0.0%) | Italy | 231 (3.0%) |
| Bulgaria | 4 (0.1%) | Algeria | 17 (0.2%) | Japan | 45 (0.6%) |
| Estonia | 11 (0.1%) | Angola | 82 (1.1%) | Kenya | 104 (1.4%) |
| Kyrgyzstan | 1 (0.0%) | Australia | 7 (0.1%) | Kuwait | 4 (0.1%) |
| Latvia | 2 (0.0%) | Austria | 2 (0.0%) | Lebanon | 2 (0.0%) |
| Lithuania | 3 (0.0%) | Bangladesh | 7 (0.1%) | Liberia | 34 (0.4%) |
| Poland | 4 (0.1%) | Belgium | 86 (1.1%) | Luxembourg | 100 (1.3%) |
| Romania | 20 (0.3%) | Benin | 29 (0.4%) | Macao | 5 (0.1%) |
| Russia | 390 (5.1%) | Brazil | 2 (0.0%) | Madagascar | 4 (0.1%) |
| Serbia | 7 (0.1%) | Burkina Faso | 8 (0.1%) | Malaysia | 16 (0.2%) |
| Slovakia | 1 (0.0%) | Burundi | 3 (0.0%) | Mali | 10 (0.1%) |
| Slovenia | 4 (0.1%) | Cameroon | 893 (11.7%) | Mauritania | 4 (0.1%) |
| Tajikistan | 1 (0.0%) | Canada | 20 (0.3%) | Morocco | 36 (0.5%) |
| Ukraine | 1 (0.0%) | Cape Verde | 90 (1.2%) | Mozambique | 11 (0.1%) |
| Uzbekistan | 2 (0.0%) | Central African Republic | 35 (0.5%) | Netherlands | 163 (2.1%) |
|  | | Chad | 14 (0.2%) | Niger | 32 (0.4%) |
|  |  | China | 37 (0.5%) | Nigeria | 1446 (19.0%) |
|  |  | Congo | 44 (0.6%) | Pakistan | 13 (0.2%) |
|  |  | Cote d'Ivoire | 17 (0.2%) | Philippines | 3 (0.0%) |
|  |  | Cuba | 122 (1.6%) | Portugal | 1417 (18.6%) |
|  |  | Cyprus | 1 (0.0%) | Republic of Korea | 51 (0.7%) |
|  |  | Czech Republic | 17 (0.2%) | Saudi Arabia | 28 (0.4%) |
|  |  | Democratic Republic of the Congo | 303 (4.0%) | Senegal | 47 (0.6%) |
|  |  | Denmark | 29 (0.4%) | Singapore | 1 (0.0%) |
|  |  | Ecuador | 3 (0.0%) | South Africa | 13 (0.2%) |
|  |  | Equatorial Guinea | 8 (0.1%) | Spain | 348 (4.6%) |
|  |  | Ethiopia | 1 (0.0%) | Sweden | 19 (0.2%) |
|  |  | Fiji | 1 (0.0%) | Switzerland | 32 (0.4%) |
|  |  | Finland | 6 (0.1%) | Taiwan | 28 (0.4%) |
|  |  | France | 265 (3.5%) | Thailand | 6 (0.1%) |
|  |  | Gabon | 27 (0.4%) | Togo | 102 (1.3%) |
|  |  | Gambia | 4 (0.1%) | Tunisia | 4 (0.1%) |
|  |  | Germany | 66 (0.9%) | Turkey | 9 (0.1%) |
|  |  | Ghana | 123 (1.6%) | Uganda | 29 (0.4%) |
|  |  | Greece | 15 (0.2%) | UK | 179 (2.3%) |
|  |  | Guinea | 7 (0.1%) | United Republic of Tanzania | 1 (0.0%) |
|  |  | Guinea-Bissau | 13 (0.2%) | U.S.A. | 75 (1.0%) |
|  |  | India | 1 (0.0%) | Venezuela | 1 (0.0%) |
|  |  | Indonesia | 2 (0.0%) | Zambia | 57 (0.7%) |
|  |  | Ireland | 1 (0.0%) | Zimbabwe | 1 (0.0%) |
|  |  | Israel | 4 (0.1%) | Unknown | 43 (0.6%) |
| **Total FSU: 453 (5.9%)** | | **Total non-FSU: 7169 (94.1%)** | | | |

**Supplementary Table S8: Country of origin of HIV-1 sequences (any genomic region) annotated as subtype G in Los Alamos database**

FSU: former Soviet Union country
